# Potential role of CSF cytokine profiles in discriminating infectious from non-infectious CNS disorders

**DOI:** 10.1371/journal.pone.0205501

**Published:** 2018-10-31

**Authors:** Danielle Fortuna, D. Craig Hooper, Amity L. Roberts, Larry A. Harshyne, Michelle Nagurney, Mark T. Curtis

**Affiliations:** 1 Department of Pathology and Laboratory Medicine, Hospital of the University of Pennsylvania, Philadelphia, Pennsylvania, United States of America; 2 Department of Neurosurgery, Thomas Jefferson University Hospital, Philadelphia, Pennsylvania, United States of America; 3 Department of Cancer Biology, Sidney Kimmel Medical College at Thomas Jefferson University, Philadelphia, Pennsylvania, United States of America; 4 Department of Pathology, Anatomy, and Cell Biology, Thomas Jefferson University Hospital, Philadelphia, Pennsylvania, United States of America; University of British Columbia, CANADA

## Abstract

Current laboratory testing of cerebrospinal fluid (CSF) does not consistently discriminate between different central nervous system (CNS) disease states. Rapidly distinguishing CNS infections from other brain and spinal cord disorders that share a similar clinical presentation is critical. New approaches focusing on aspects of disease biology, such as immune response profiles that can have stimulus-specific attributes, may be helpful. We undertook this preliminary proof-of-concept study using multiplex ELISA to measure CSF cytokine levels in various CNS disorders (infections, autoimmune/demyelinating diseases, lymphomas, and gliomas) to determine the potential utility of cytokine patterns in differentiating CNS infections from other CNS diseases. Both agglomerative hierarchical clustering and mixture discriminant analyses revealed grouping of CNS disease types based on cytokine expression. To further investigate the ability of CSF cytokine levels to distinguish various CNS disease states, non-parametric statistical analysis was performed. Mann-Whitney test analysis demonstrated that CNS infections are characterized by significantly higher CSF lP-10/CXCL10 levels than the pooled non-infectious CNS disorders (p = 0.0001). Within the infection group, elevated levels of MDC/CCL22 distinguished non-viral from viral infections (p = 0.0048). Each disease group of the non-infectious CNS disorders independently showed IP-10/CXCL10 levels that are significantly lower than the infection group [(autoimmune /demyelinating disorders (p = 0.0005), lymphomas (p = 0.0487), gliomas (p = 0.0294), and controls (p = 0.0001)]. Additionally, of the non-infectious diseases, gliomas can be distinguished from lymphomas by higher levels of GRO/CXCL1 (p = 0.0476), IL-7 (p = 0.0119), and IL-8 (p = 0.0460). Gliomas can also be distinguished from autoimmune/demyelinating disorders by higher levels of GRO/CXCL1 (p = 0.0044), IL-7 (p = 0.0035) and IL-8 (p = 0.0176). Elevated CSF levels of PDGF-AA distinguish lymphomas from autoimmune/demyelinating cases (p = 0.0130). Interrogation of the above comparisons using receiver operator characteristic analysis demonstrated area under the curve (AUC) values (ranging from 0.8636–1.0) that signify good to excellent utility as potential diagnostic discriminators. In conclusion, our work indicates that upon formal validation, measurement of CSF cytokine levels may have clinical utility in both identifying a CNS disorder as infectious in etiology and, furthermore, in distinguishing viral from non-viral CNS infections.

## Introduction

Rapid identification of CNS disease is critical to implement prompt measures and initiate appropriate disease-specific treatments [1–5]. CNS infections are major causes of morbidity and mortality, yet such infections may be amenable to therapeutic intervention if promptly diagnosed [6]. Given that many pathogens worldwide are rapidly expanding their geographic ranges, certain infections may not be considered at the initial clinical presentation, creating a lag between clinical suspicion and diagnosis [7]. Continuous advancement in therapeutic and preventive options, such as small molecule antiviral drugs, therapeutic antibodies, and new vaccines, makes early diagnosis of infection even more important [1]. Initial identification of a CNS disorder as infectious and distinguishing it from other non-infectious pathologic processes, however, is not entirely straightforward. The differential diagnoses considered in a patient with symptoms of CNS disorder (e.g., acute mental status change, focal neurologic deficit, severe headache, and photophobia) are numerous and often include infection, autoimmune disorders, demyelinating disease and neoplasms [4,8,9]. Importantly, therapy for these various diseases is drastically different [3,4,10–12]. For example, immunosuppressive and immunomodulatory agents indicated in CNS autoimmune disease and demyelinating disorders (including multiple sclerosis) would be contraindicated and potentially detrimental in the setting of a CNS infection.

Due to its close anatomical relationship to critical structures in the CNS, the CSF serves as a convenient conduit for inflammatory mediators and signaling proteins released during changes in the CNS environment. The rapidly responding innate immune system is present and active in the CNS and is sensitive to a variety of alterations in CNS homeostasis. CSF, therefore, can be examined to understand the current state of immune activation. When activated in response to infection, tissue damage, or mass lesions, pro- and anti-inflammatory cytokines and growth factors are released and detectable in the CSF [13–19]. It is known that pathogens, autoimmune processes, demyelination, and neoplasms target different CNS resident cells and activate innate immunity in different ways, resulting in distinct patterns of CSF cytokines which reflect the range of pathologies [13,18–22].

Pattern recognition receptors, such as toll-like receptors (TLRs), Nod-Like receptors (NLRs), and RIG-like receptors (RLRs), play a crucial role in responding to various insults and generating the innate response [23,24]. The diversity and subsequent combinations of pattern recognition receptors situated in various cell types and subcellular compartments allows for detection of a wide array of cellular danger signals and ligands, resulting in the release of cytokines and induction of fairly customized inflammatory and anti-inflammatory responses. In view of this, the present study involving the quantification of CSF cytokine levels and subsequent statistical analysis was performed to investigate cytokine profiles of different CNS disease states and test their potential to differentiate distinct neuropathologic processes.

## Methods

### Populations

This retrospective study was conducted at Thomas Jefferson University Hospital (TJUH), Philadelphia, PA and was approved by the TJUH institutional review board (Thomas Jefferson University, Office of Human Research, Division of Human Subjects Protection Institutional Review Board Control #15D.032) pursuant to title 45 code of United States of America Regulations Part 46.116(d). The CSF samples analyzed were de-identified and no longer needed for clinical analysis. The samples were originally obtained by lumbar puncture as part of a clinical work-up of patients prior to specimen de-identification, and all samples were obtained with appropriate consent by the clinical team during the clinical evaluation. Personal data of patients was protected at all times. Criteria for inclusion were patients at TJUH age 2 to 80 years of age with CNS disease. CSF laboratory studies, such as white blood cell (WBC) count, glucose concentration, and protein levels, were a component of the patient’s clinical evaluation. Samples selected for cytokine analysis included seven control patients negative for neuro-inflammatory processes, 15 patients with CNS infections, three patients with malignant glial (astrocytic) neoplasms, 11 patients with autoimmune and demyelinating disease (autoimmune/DM), and six patients with B-cell lymphoma involving the CNS.

### Cytokine analysis

All CSF samples (n = 43) were analyzed using the human Cytokine/Chemokine Magnetic Bead Panel Millipore plates on a Luminex FlexMPA 3D for the following analytes: epidermal growth factor (EGF), fibroblast growth factor 2 (FGF2), eotaxin/CCL11, transforming growth factor alpha (TGF-α), granulocyte-colony stimulating factor (G-CSF), macrophage derived chemokine (MDC/CCL22), granulocyte macrophage colony-stimulating factor (GM-CSF), interferon-γ (IFN-γ), GRO/CXCL1, MCP3/CCL7, IL12p40, MCP-1/CCL-2, MIP1-α/CCL3, MIP1-β/C CL4, tumor necrosis factor-α (TNF-α), tumor necrosis factor-β (TNF-β), IL-12p70, Fractalkine/CX3CL1, IL-1α, IL-1β, IL-2, IL-4, IL-3, IL-5, IL-6, IL-7, IL-8, IL-9, IL-10, IL-13, IL-15,IL-17α, IL-1Ra, IFN-α2, IP-10/CXCL10, sCD40L, FLT-3L, vascular endothelial growth factor (VEGF), platelet-derived growth factor AA (PDGF-AA), PDGF-AB/BB, and RANTES. Samples were analyzed in duplicate by a FlexMAP 3D (Luminex). Standard curves were generated for each cytokine, and median fluorescent intensities were transformed into concentrations by 5-point, non-linear regression. Data was exported to a Microsoft Excel file.

### Statistical analyses

Statistical analyses including agglomerative hierarchical analysis, discriminant analysis (DA), and principal component analysis (PCA) were performed using the XLStat statistics program. These methods help assess the relationships of cytokine profiles based in the innate immune response reflected by CSF cytokine levels. DA was performed to determine informative cytokines. Informative cytokines were then used for generation of three-dimensional PCA plots to identify disease-type clustering as a function of informative cytokine levels. The Mann-Whitney test for univariate, non-parametric analysis using the Prism GraphPad Statistics Program was applied for comparison of cytokines among the disease groups (controls, infections, gliomas, autoimmune/DM, lymphomas). Receiver operator characteristic (ROC) analysis was performed using Prism GraphPad Statistics Program.

## Results

### Patient demographics, including age, sex, and diagnosis

CSF samples from 43 patients were included, spanning a wide range of CNS diseases: various infections (viral, bacterial, fungal and protozoan), autoimmune and demyelinating diseases, lymphomas, and gliomas. A variety of different pathogens was included in the infectious group to reproduce the common clinical scenario in which a range of pathogens are in the differential diagnosis to exclude infection. The control samples are from non-infectious cases with a thorough, negative clinical work-up, and include diagnoses such as idiopathic intracranial hypertension, headache, and hydrocephalus. The diagnosis, patient age, and sex for each case are presented in Table 1. The median age of the control group is 50 years. The cases include seven controls, 15 infectious cases (three fungal, seven viral, five bacterial, and one protozoan), three malignant astrocytic glioma cases, 12 autoimmune/demyelinating cases, and six cases of B-cell lymphomas involving the CNS (four primary CNS lymphomas of diffuse large B-cell type and two systemic B-cell lymphomas involving the CNS). Additional clinical information, including co-morbid diagnoses, is presented in supplementary documents (S1 Table, S2 Table, S3 Table and S4 Table).

**Table 1:** Disease class, specific diagnosis, age, and sex for cases (CSF samples).

Disease Class	Diagnosis	Age (years)	Gender
Control	Headache	50	F
Control	Transient ischemic attack	77	F
Control	Idiopathic intracranial hypertension	40	F
Control	Headache	55	F
Control	Hydrocephalus/VP shunt	51	F
Control	Headache	33	F
Control	Hydrocephalus/VP Shunt	2	M
Infection	Cryptococcal meningitis/HIV	54	M
Infection	Enterovirus meningitis	19	M
Infection	JC virus/PML/HIV	46	M
Infection	HPeV encephalitis	17	F
Infection	Cryptoccal meningitis post heart transplant	55	M
Infection	WNV encephalitis	33	M
Infection	Lyme disease	48	M
Infection	Toxoplasmosis/status post chemotherapy	73	F
Infection	Cryptococcal meningitis/HIV	42	M
Infection	TB meningitis post adalimumab therapy	30	F
Infection	*Staphylococcus epidermidis* meningitis/HIV	32	M
Infection	*Streptococcus mitis* meningitis	73	M
Infection	Viral meningitis, not otherwise specificied	20	F
Infection	TB meningitis/HIV	35	F
Infection	JCV meningitis/immunosuppression for lupus	52	F
Glioma	Anaplastic astrocytoma	77	F
Glioma	Recurrent glioblastoma	60	M
Glioma	Recurrent glioblastoma	50	F
Autoimmune/DM	Autoimmune encephalopathy	53	F
Autoimmune/DM	Post-viral cerebellitis	51	F
Autoimmune/DM	Transverse myelitis	80	F
Autoimmune/DM	CNS vasculitis	27	F
Autoimmune/DM	Paraneoplastic cerebellar dysfunction	55	M
Autoimmune/DM	Acute disseminated encephalomyelitis	39	M
Autoimmune/DM	Acute disseminated encephalomyelitis	80	M
Autoimmune/DM	Anti-acetylcholine ganglionic neuronal receptor autoimmune encephalopathy	66	F
Autoimmune/DM	Multiple sclerosis	29	F
Autoimmune/DM	Multiple sclerosis	30	F
Autoimmune/DM	Multiple sclerosis	46	M
Autoimmune/DM	Tumefactive multiple sclerosis	30	F
Lymphoma	Primary CNS Lymphoma	21	M
Lymphoma	Primary CNS Lymphoma	72	M
Lymphoma	Primary CNS Lymphoma	58	F
Lymphoma	Primary CNS Lymphoma	78	M
Lymphoma	Systemic DLBCL involving CNS	69	F
Lymphoma	Systemic Burkitt lymphoma involving CNS	37	M

### CSF laboratory findings and parameters by CNS disease

The initial CSF parameters routinely measured in patients with suspected CNS disease include CSF WBC count (cells/μl), CSF protein concentration (mg/dl), and CSF glucose concentration (mg/dl). Summaries of these findings are shown in Table 2. Using Mann-Whitney tests of significance, it was found that the protein levels in the infection group and the glioma group are statistically higher than the protein levels in the control group. Other than these two statistical differences, with the numbers of samples available for analysis, there were no other significant differences between any of the CNS disease groups for any of the CNS parameters listed above.

**Table 2:** Patient age and CSF WBC count, protein concentration, and glucose data.

	Controls	Infections	Gliomas	Autoimmune/Demyelinating	Lymphomas
*n*	7	15	3	12	6
**Age, years**	50(2–77)	39(17–73)	60(50–77)	51(27–88)	64(21–78)
**CSF WBC(cells/μl) (normal = 0)**	2(0–2)	29(0–511)	7(0–263)	9(0–119)	10(0–25)
**CSF Protein (mg/dl) (normal range = 15–55)**	33(16–47)	70*(19–910)	124* (50–133)	47(14–183)	47(25–105)
**CSF Glucose (mg/dl) (normal range = 40–70)**	68(32–82)	60(32–87)	66(11–89)	64(42–197)	78(50–95)

Values for age, CSF WBC, CSF Protein, and CSF Glucose are medians (minimum-maximum).

*The CSF protein levels in the infection group and the glioma group were statistically different from the CSF proteins levels in the control group (p = 0.0143 and p = 0.0163 respectively).

### Heat map and agglomerative hierarchical clustering of cytokine levels demonstrate three major classes of CNS disease based on cytokine expression

**Fig 1:**
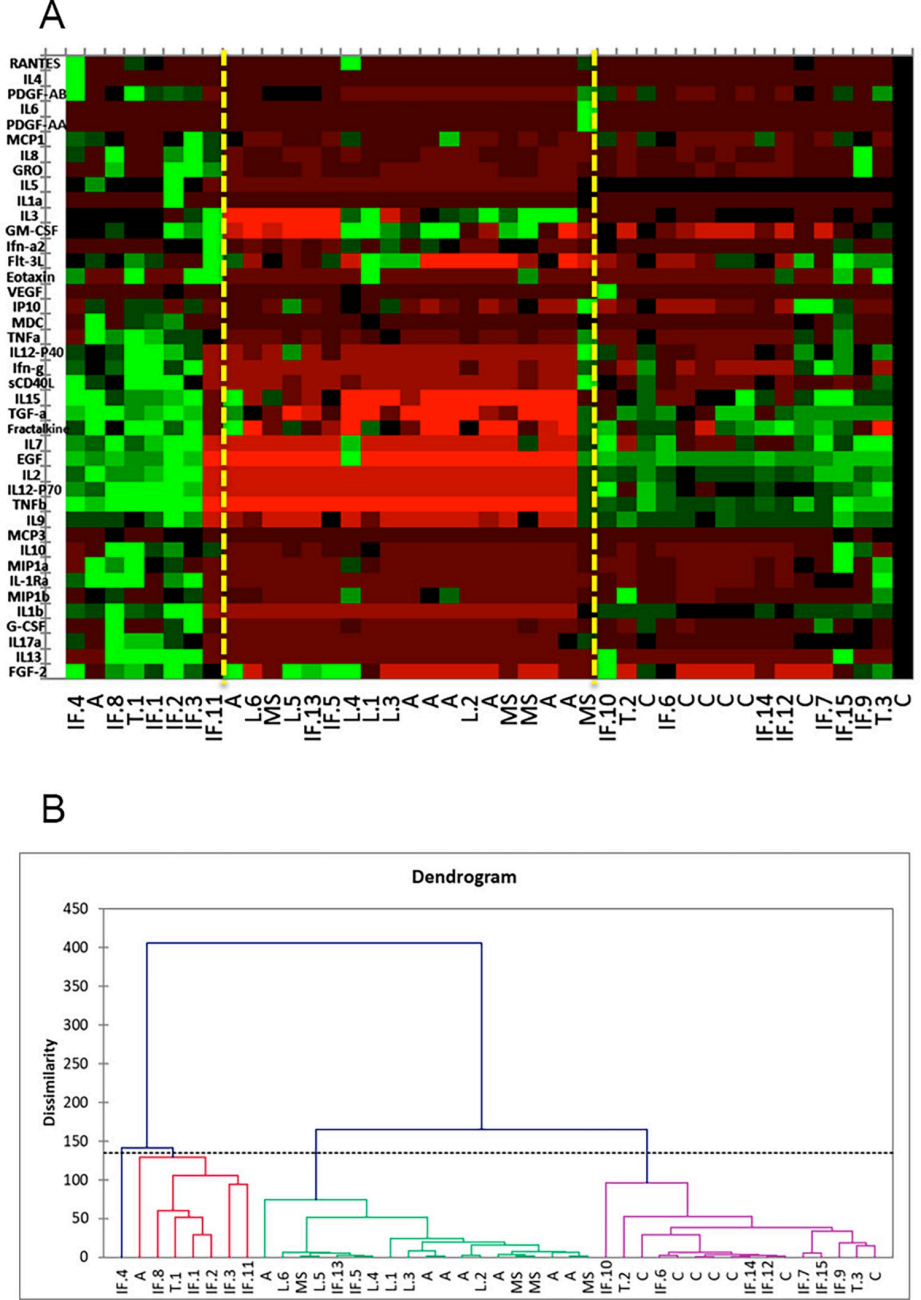
Heat map and dendrogram based on agglomerative hierarchical clustering (AHC). (A) The heat map visually depicts the cytokine levels using colors (red = low, green = high) shown on the right. The heat map was created in conjunction with agglomerative hierarchical clustering (AHC). The three classes shown in the AHC are reflected here. Vertical, yellow lines have been drawn to aid in this separation of classes. C = control; L = CNS B-cell lymphoma; IF = infection, T = tumor (high grade gliomas), A = autoimmune; MS = multiple sclerosis. (B) The AHC based dendrogram includes all 43 cases and shows separation of CNS diseases into three major classes demonstrating a trend toward clustering of disease types based on overall cytokine profiles. See supplementary table (S5 Table) for a key to the abbreviations of the disease type/class used in the heat map and dendrogram.

A heat map and dendrogram were generated using all 43 patient samples with the measured levels from 41 cytokines: epidermal growth factor (EGF), fibroblast growth factor 2 (FGF2), eotaxin/CCL11, transforming growth factor alpha (TGF-α), granulocyte-colony stimulating factor (G-CSF), macrophage derived chemokine (MDC/CCL22), granulocyte macrophage colony-stimulating factor (GM-CSF), interferon-γ (IFN-γ), GRO/CXCL1, MCP3/CCL7, IL12p40, MCP-1/CCL-2, MIP1-α/CCL3, MIP1-β/CCL4, tumor necrosis factor-α (TNF-α), tumor necrosis factor-β (TNF-β), IL-12p70, fractalkine/CX3CL1, IL-1α, IL-1β, IL-2, IL-4, IL-3, IL-5, IL-6, IL-7, IL-8, IL-9, IL-10, IL-13, IL-15, IL-17α, IL-1Ra, IFN-α2, IP-10/CXCL10, sCD40L, FLT-3L, vascular endothelial growth factor (VEGF), platelet-derived growth factor AA (PDGF-AA), PDGF-AB/BB, and RANTES (Fig 1). These analytical tools help to visualize large sets of data to assess for overarching trends and patterns. The heat map (Fig 1A) displays all 43 cases with the corresponding CSF cytokine levels represented by the color scale (red = low, green = high). Each column in the graph represents a case. By overall cytokine levels, we can see that the cases separate into major classes based on three predominant cytokine profiles. Two vertical lines (yellow) are superimposed on the heat map to further illustrate these classes.

The class distinctions demonstrated by the heat map correspond directly to the classes depicted in the dendrogram (Fig 1B). Agglomerative hierarchical clustering (AHC) was generated from data expressed by the heat map. AHC is based on Ward’s method calculation to minimize the variance within each cluster. Successive clustering progresses in a “bottom-up” approach in order to create homogenous classes or clusters of diseases arranged graphically in the form of a dendrogram.

The dendrogram (Fig 1B) includes all 43 cases and shows separation of these cases into three broad classes. One class contains the vast majority of the autoimmune/DM cases and all lymphoma cases. The few CNS infections included in this class are in severely immunosuppressed patients with immunosuppression-related infections: two cases of JC virus progressive multifocal leukoencephalopathy (PML) in a patient with a heart transplant and in a patient with human immunodeficiency virus (HIV), and one case of neurotuberculosis in a patient with HIV. On the heat map, this class shows an overall subdued cytokine pattern, displaying the least relative increases in cytokine levels (this is indicated by the predominance of red colors on the graph).

The second class on the dendrogram includes WHO grade IV malignant astrocytic neoplasms; CNS fungal infections (cerebromeningeal cryptococcus infection); CNS viral infections (West Nile virus and human parechovirus meningitis); CNS protozoan infections (Toxoplasmosis); and the control cases. On the heat map, this class generally shows intermediate cytokine levels, ranging between the levels observed in the first class and the third class.

The third class corresponds to diseases with the most pronounced increase of cytokine levels shown on the heat map, which includes eight cases: a fatal case of neurotuberculosis in a patient treated with adalimumab; enterovirus (EV) meningitis; three cases of bacterial meningitis (*Streptococcus mitis*, *Borrelia burgdorferi* (Lyme disease), *Staphylococcus epidermidis*); a case of cryptococcal meningitis; one case of anti-acetylcholine ganglionic neuronal receptor autoimmune encephalopathy; and a WHO grade III malignant astrocytic neoplasm.

This initial assessment of the entire data set with the heat map and dendrogram supports further investigation of our hypothesis that CNS diseases can be partitioned based on a composite cytokine innate immune profile in a reproducible manner. For example, all six CNS lymphoma cases were present in the same class as 11 of the 12 autoimmune/DM cases, along with all of the severely immunosuppressed patients with CNS infections. The grouping of a disease type within a class confirms that the innate immune response, as reflected by cytokine levels, is relatively similar within a disease class. Additionally, the grouping of more than one disease type within a given class suggests that certain disease states may share some characteristics of innate immune response (i.e. CNS lymphomas and autoimmune/DM disorders).

### A panel of informative cytokines reveals a pronounced separation of disease classes based on cytokine levels

In order to achieve the most efficient differentiation of CNS diseases based on CSF cytokine levels, we used a combination of statistical methods to identify a panel of informative cytokines which allow for a more precise separation of the different disease states. Using all 41 cytokines, discriminant analysis was applied. Discriminant analysis is a method used to statistically verify whether groups (here, CNS disease states) can be classified based on measured characteristics (here, cytokines). It can also be used to isolate the variables which have the greatest impact on the separation of the groups. Based on the application of a chi square test, the p-value generated for each cytokine (variable) signifies its contribution to the separation of the cases and the groups of diseases.

**Fig 2:**
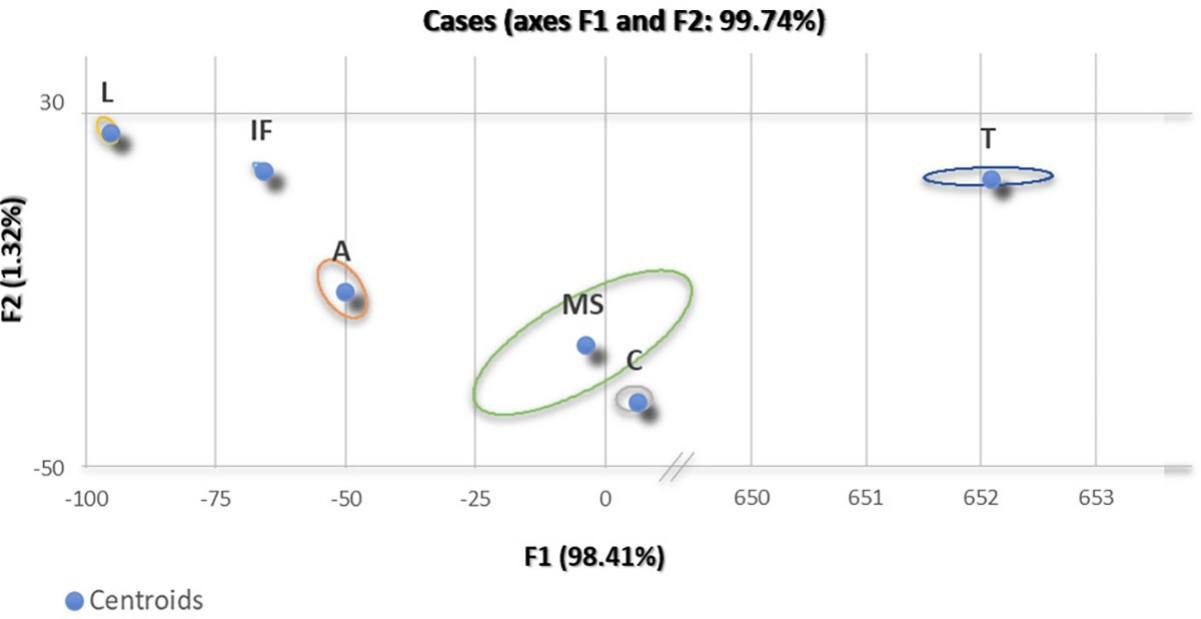
Discriminant analysis observation plot. According to discriminant analysis, 100% of our 43 cases were assigned appropriately to their respective disease groups (infection, autoimmune, tumor, DM, control).

According to discriminant analysis, 100% of our 43 cases were assigned appropriately to their respective disease groups (infection, autoimmune, demyelination (DM), tumor, lymphoma, and control). Fig 2 shows the observations plotted on the factor axes. The plot shows the groups’ centroids with a surrounding ring demonstrating the distribution of the observations within each disease group. This plot demonstrates that CNS diseases separate well based on the cytokine expression.

Utilizing a combination of the aforementioned methods, a panel of cytokines was selected from the initial 41 cytokines: EGF, MDC/CCL22, PDGF-AA, Fractalkine/CX3CL1, IFN-γ, GRO/CXCL1, IL-1β, IL-2, IL-7, IL-8, IL-9, IP-10/CXCL10, TGF-α, IL12-p40, IL12-p70, IL13, IL-15, and TNF-β. Principal component analysis (PCA) is another statistical technique used to assess patterns and correlations in a data set. This method transforms a multi-dimensional set of data to a practical dimension for viewing data trends on a plot. PCA differs from the discriminant analysis in that PCA constructs the best clustering and discrimination of the observations without any previous knowledge of any predetermined group allocations. The generated principal components (P1, P2, P3, etc.) are linear representations of the variables (cytokines) which describe the maximum variation in the data set.

By plotting all cases using coordinates generated by the principal components and the observed values, one can visualize the multi-dimensional (in our case 18-dimensional) data set on a three-dimensional plot. Here, approximately 70% of the variance in our data can be explained by the first three principal components. While a common use of PCA is to demonstrate similarity of observations and of variables as points on maps as a step in the validation of methods, we generated a PCA plot using our original data set to demonstrate what analysis of a larger data set might yield during a future validation of our proposed approach (S1 Fig).

### Mann-Whitney test analysis identifies cytokines helpful in distinguishing CNS disorders

**Fig 3:**
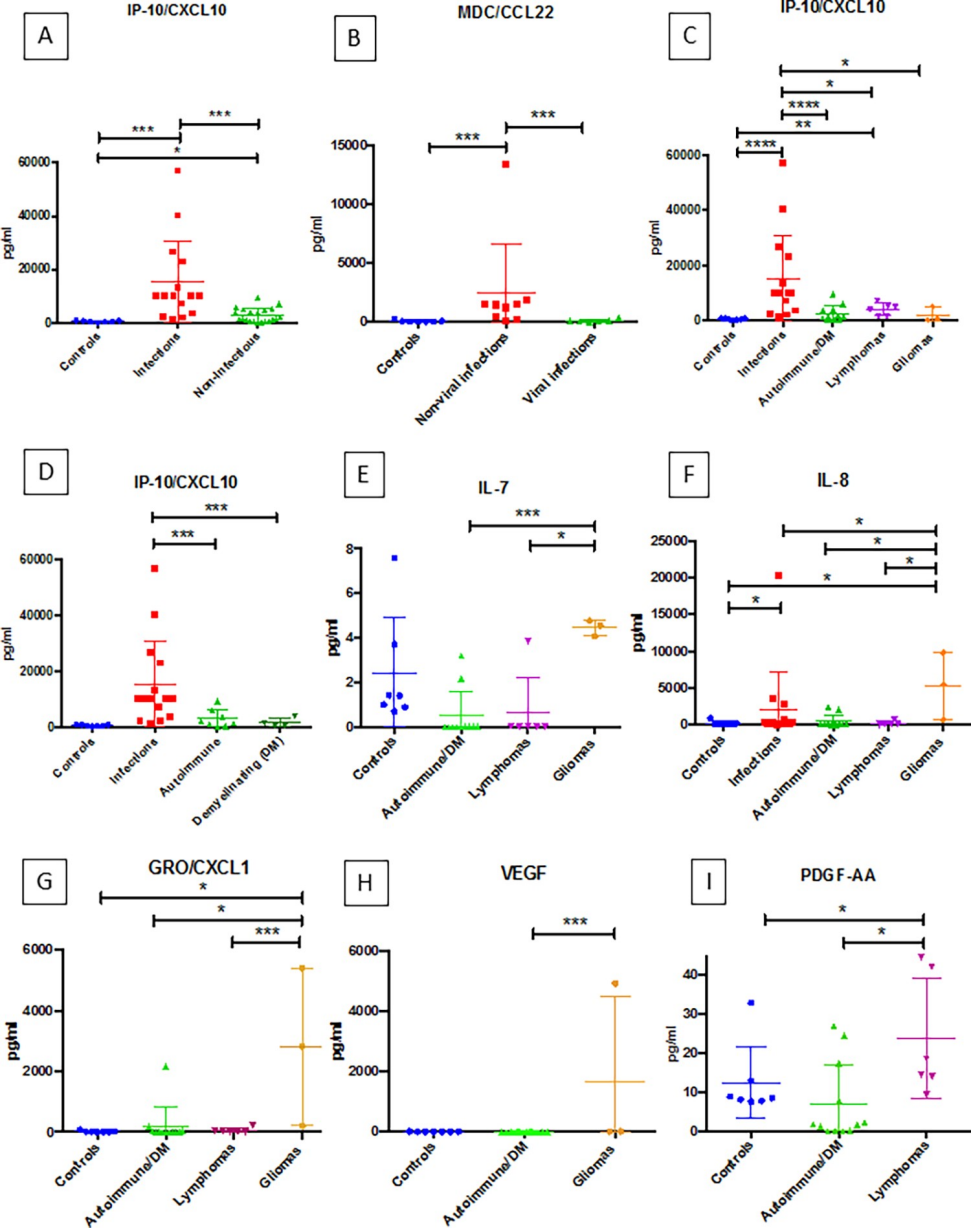
**(A-I): Results from mann-whitney analyses for specific cytokines (IP-10/CXCL10, MDC/CCL22, IL-7, IL-8, GRO/CXCL1, VEGF, and PDGF-AA).** Each graph shows the cytokine level distribution for the respective disease groups. In each graph, horizontal lines with an asterisk (*) indicate the presence of statistically significant differences between groups for the given cytokine. The number of asterisks corresponds to the calculated p-value (* = p < 0.05, ** = p < 0.01, *** = p < 0.005, **** = p < 0.001).

To assess the potential utility of quantifying levels of individual CSF cytokines in distinguishing between distinct CNS diseases, we used the Mann-Whitney test of significance to test for statistical significance in cytokine levels between various CNS disease groups. IP-10/CXCL10 levels were significantly higher in the pooled infectious cases compared to the pooled non-infectious cases (p < 0.0001) and controls (p < 0.0001) (Fig 3A). Infections of various types are pooled in our analysis, since a variety of CNS infections can present to clinical attention with indistinguishable symptoms and similar results in initial testing.

Two critical questions to be answered in the clinical setting that represent major branch points in the clinical decision making process are as follows: *“Is this disease an infection*?*”* and *“If the disease is an infection*, *is the pathogen a virus or a non-viral pathogen*?*”* Analysis of IP-10 levels (Fig 3A) may provide information relative to the likelihood of whether the process is an infection. Within the infectious group, MDC/CCL22 levels were significantly higher in non-viral infections compared to viral infections (p = 0.0048) and controls (p = 0.0012) (Fig 3B). Thus, CSF measurement of IP-10/CXCL10 levels may be useful in identifying a CNS disease state as suspicious for infection with further stratification of the disease using MDC/CCL22 levels into viral versus non-viral infection subtypes. Levels of IP-10/CXCL10 were also significantly higher in the infection group when compared to the specific autoimmune/DM disease cases (p = 0.0005), lymphoma (p = 0.0487), and glioma (p = 0.0294) groups, and IP-10/CXCL10 levels were significantly higher in infectious and lymphomas compared to controls (p < 0.0001 and p = 0.0012, respectively) (Fig 3C). Elevated levels of IP-10/CXCL10 were also significantly higher in infectious cases than both autoimmune cases and demyelinating cases when these two groups were analyzed separately (Fig 3D).

Interrogation of other cytokines seen in Fig 3 demonstrated significant differences between non-infectious disease states to potentially contribute to the characterization of the cases not suspected of being infectious. IL-7, IL-8, GRO/CXCL1 and VEGF were informative in distinguishing WHO grade III and IV gliomas from the other disease states studied (Fig 3E–3H). IL-7 levels were significantly higher in gliomas compared to autoimmune/DM cases (p = 0.0035) and lymphomas (p = 0.0119). Gliomas also displayed higher IL-8 levels when compared to infections (p = 0.0392), autoimmune/DM cases (p = 0.0176), lymphomas (p = 0.0460), and controls (p = 0.0333). GRO/CXCL1 levels were higher in gliomas compared to autoimmune/DM (p = 0.0044), lymphomas (p = 0.0476) and controls (p = 0.0167). Higher levels of VEGF are seen in gliomas compared to autoimmune/DM cases (p = 0.0286). While lymphomas and autoimmune/DM cases appear as a single class on AHC, PDGF-AA levels prove helpful in separating these two disease groups with CSF from patient with lymphomas having significantly higher levels than autoimmune/DM cases (p = 0.0130) and controls (p = 0.0221) (Fig 3I). For the analytes presented in Fig 3 (IP-10/CXCL10, MDC/CCL2, IL-7, IL-8, GRO/CXCL1, VEGF and PDGF-AA), a level of significance of p < 0.05 or smaller signifies an observed power of greater than 50%.

### ROC Analysis demonstrates strong potential of cytokine levels for distinguishing CNS disorders

**Fig 4:**
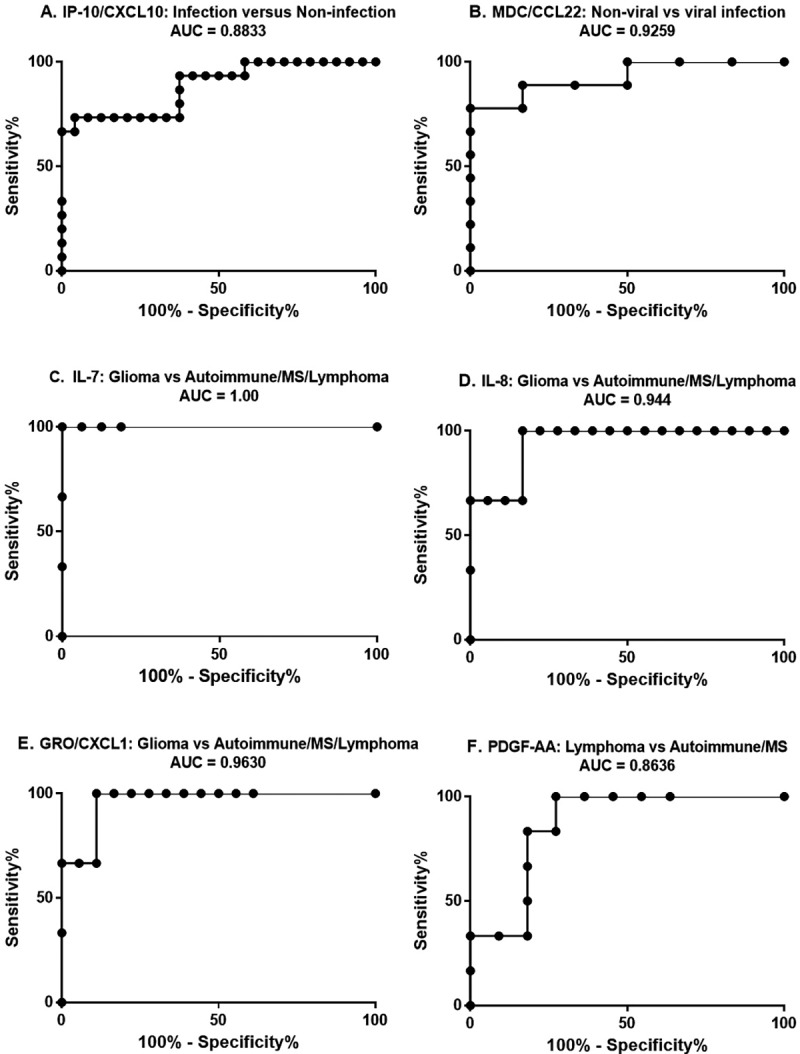
**(A-F): ROC curves for IP-10/CXCL10 (A), MDC/CCL22 (B), IL-7 (C), IL-8 (D), GRO/CXCL1 (E), and PDGF-AA (F).** The title of each graph includes the groups that were compared, as well as the respective AUC.

**Fig 5:**
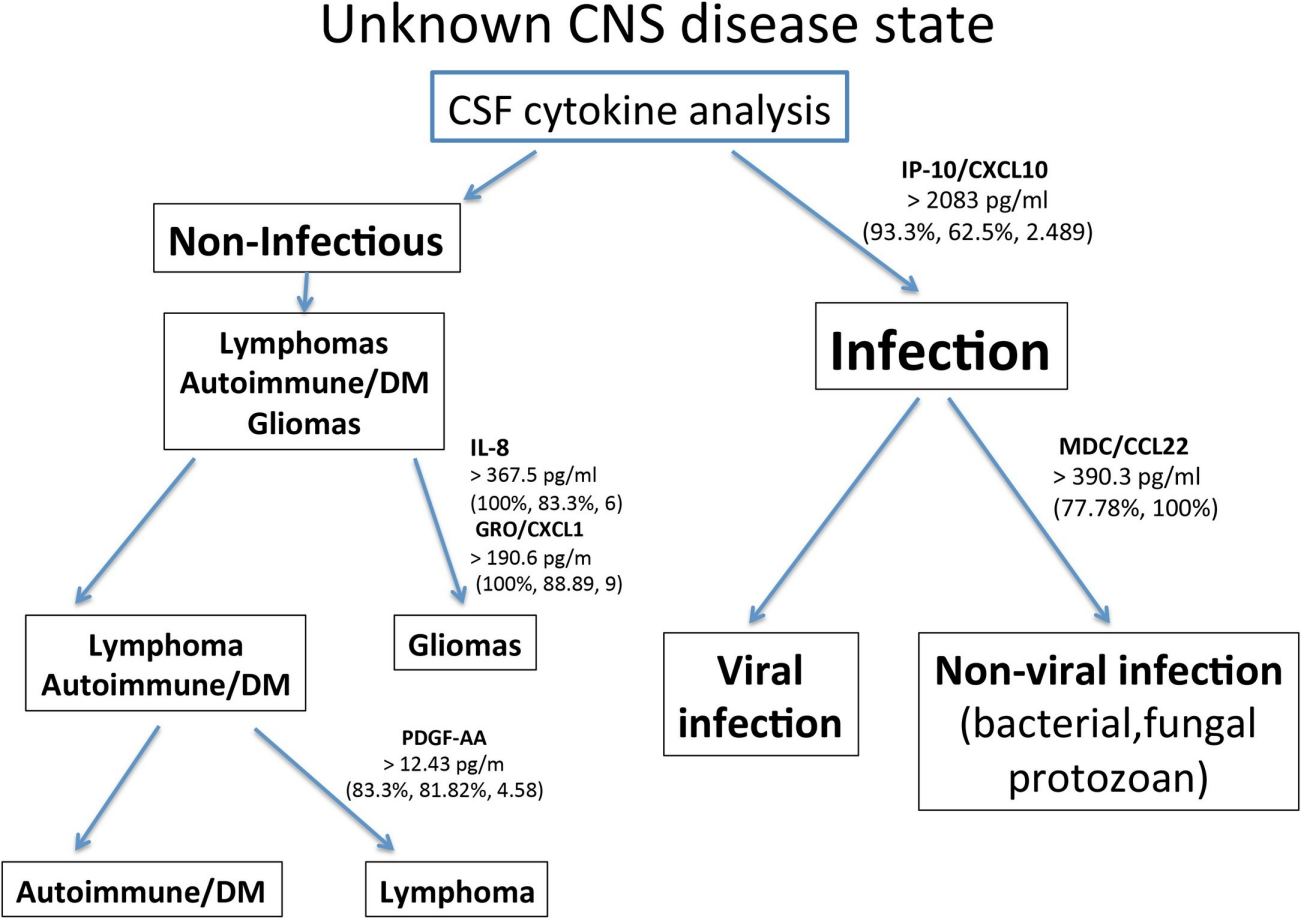
Proposed algorithm for diagnosis of CNS diseases using a selective cytokine panel. The cytokines included in the algorithm include IP-10/CXCL10, MDC/CCL22, IL-8, GRO/CXCL1, and PDGF-AA. Potential “cut-off” values for interpretation are presented here. Sensitivity, specificity, and likelihood ratios (if available) corresponding to the cut-off values have been generated from the ROC analyses and are also featured.

Receiver operator characteristic (ROC) curve analysis is a tool to explore the inherent utility of a method or assay as a diagnostic test. Here, we used ROC to interrogate the potential utility of the above cytokines as individual tests. ROC curves with the corresponding AUC for IP-10/CXCL10, PDGF-AB/BB, IL-7, IL-8, GRO/CXCL1, and PDGF-AA are shown in (Fig 4A–4F). All of the AUC values ranged between 0.8000 and 1. AUC values in this range are considered to be in either the good (0.8–0.9) or excellent (0.9–1.0) range when grading test adequacy. The results of this analysis support the potential of using levels of these cytokines in CSF to distinguishing different CNS disorders. ROC analysis also suggests analyte cut-off values along with corresponding sensitivities and specificities. Based on the cut-off values suggested by ROC, we constructed a prototype diagnostic algorithm flowchart (Fig 5) using the different CSF cytokine levels to identify probable infectious cases, sub-classify them as viral or non-viral, and suggest the nature of the non-infectious cases. Such an approach may potentially be used in the clinical setting following validation of a larger data set. The cytokine data and the CSF count, CSF glucose, and CSF protein data are available for review in a supporting information file (S1 Data).

## Discussion

The Luminex FlexMPA 3D technology utilized in our study to quantify CSF cytokine levels uses commercially available laboratory instruments and reagents. This method combines immunoassay principles and fluorescent-coded beads in a flow cytometry-based system to detect and quantitate cytokine levels. Multiple cytokines can be quantified in a single sample of 50–100μl. The data presented suggests that such quantification of CSF cytokines may be useful in contributing to the identification of CNS disease class present in a patient.

One of the most frequent challenges in the evaluation of CNS disorders for therapeutic intervention is the considerable overlap in clinical presentation of a wide variety of diseases. The identification of a CNS disease process as infectious is a critical and common clinical conundrum. CNS infections are caused by an incredibly diverse group of pathogens, and the consequences of delaying early intervention or choosing an inappropriate treatment can have significant adverse clinical consequences. What is indicated for one disease class may be contraindicated in another [25].

The goal of the current study was to determine if assessing CSF cytokine levels may be able to facilitate the rapid identification of a CNS disease process as infectious, distinguish between viral and non-viral infections, and also contribute to the general classification of the diseases process if non-infectious in etiology. The present study demonstrates that in a clinical situation with unclear etiology, analysis of CNS cytokine levels may help determine if the CNS disorder is infectious, and if not, provide a likelihood of the particular non-infectious disease process. In this study, multiple infectious agents are pooled in a single group to reproduce the clinical setting where a range of infectious agents can present with similar symptoms and initial lab results.

Determination of IP-10/CXCL10 CSF levels provides an important early branch point in the proposed prototype diagnostic algorithm (Fig 5), in which elevated IP-10/CXCL10 levels rapidly identify a case as a probable CNS infection. Further cytokine level-based subclassification of an infectious case is done by assessing the MDC/CCL22 levels. High MDC/CCL22 levels suggest a non-viral infectious agent rather than a viral pathogen. In the non-infectious arm of this algorithm (low IP-10/CXCL10 levels), elevated levels of IL-8 and GRO/CXCL1 favor a diffuse glioma. Autoimmune/DM and CNS B-cell lymphoma can be further delineated by using PDGF-AA. Sample cut-off values (diagnostic thresholds) generated by the ROC analyses are featured in the proposed algorithm.

Each cytokine we have identified as potentially useful in the diagnostic discrimination of CNS diseases has been discussed in the literature and is associated with various aspects of these disease processes. IP-10/CXCL10, the cytokine we identify as useful in distinguishing infectious from non-infectious CNS processes, is released from a variety of cell types in response to IFN-γ, a major cytokine in the innate response to infection. IP-10/CXCL10 is involved in coordinating critical components of the inflammatory cascade, including chemotaxis, apoptosis, and inhibition of cell growth [26]. IP-10/CXCL10 has been detected in various body fluids in bacterial, viral, fungal, and parasitic infections [27–32]. MDC/CCL22, the cytokine we identify as potentially useful in distinguishing viral from non-viral infection, is involved in chronic inflammation, lymphocyte and dendritic cell homing, and in the overall modulation of innate immunity during infections [33]. Elevated MDC/CXCL22 levels have been documented in bacterial infections and are present in the response to both fungal and protozoan pathogens [34–37]. Our observations suggest elevated levels of IL-7, IL-8, and GRO/CXCL1 are useful in identifying a CNS disease process as a diffuse glial neoplasm. IL-7, IL-8, and GRO/CXCL1 have each been implicated in glioma biology [38–41]. Although we demonstrate that autoimmune/DM cases and CNS B-cell lymphomas share a remarkably similar CSF cytokine profile, CSF PDGF-AA levels can distinguish these two disease processes. PDGF-AA is a growth factor that promotes angiogenesis and is produced by vascular endothelial cells, fibroblasts, smooth muscle cells, and lymphoma cells [42,43]. PDGF-AA has been shown to be involved in angiogenesis in mantle cell lymphomas, and increases in PDGF-AA gene expression and protein levels have been reported in diffuse large B cell lymphomas, including EBV-positive B-cell lymphomas [44–46].

The data provided, including the heat map and dendrogram generated using all 41 cytokines, demonstrates that different diseases initiate distinct CNS innate immune responses. This finding is logical given that the innate immune system is known to tailor its response to some degree to the pathologic stimulus [20]. Different types of infectious pathogens, tumors, and states of immune dysregulation activate different sets of pattern recognition receptors leading to the release of different arrays of innate immune effectors [24]. Specifically, of the 41 cytokines analyzed, IP-10/CXCL10, MDC/CCL22, IL-8, GRO/CXCL1, and PDGF-AA were found to contribute significantly in distinguishing disease types, and these five analytes form the basis of our potentially useful diagnostic algorithm (Fig 5).

## Conclusion

We show that determination of CSF cytokine levels provides information about the current state of the CNS at the time the CSF sample is obtained. This finding suggests that upon formal validation, quantification of CSF cytokine levels may have clinical application for the rapid identification of CNS disease as infectious versus non-infectious, as well as beyond this initial distinction. A goal of our proposed algorithm is to guide clinical decisions using the likelihood and probability that a given disease process is present based on the cytokine profile. The patient samples analyzed represent a broad range of disease processes taken at different times in the disease course. This wide sampling is representative of the clinical spectrum of both disease and sample collection timing. Even with our current sample size, the trends in cytokine levels we observe are statically significant and allow for identification of cytokine profiles useful in discrimination among CNS disease states. Further development and validation of this approach may lead to an improved method to rapidly distinguish infectious from non-infectious CNS disease processes.

## Supporting information

S1 Table Disease class, diagnosis, Co-morbidities, age and sex for cases. Information regarding pre-existing medical conditions of the patients from whom the CSF samples were obtained including control and infection cases.
(TIFF)

S2 Table Disease class, diagnosis, Co-morbidities, age and sex for cases. Information regarding pre-existing medical conditions of the patients from whom the CSF samples were obtained including infection, glioma and autoimmune/DM cases.
(TIFF)

S3 Table Disease class, diagnosis, Co-morbidities, age and sex for cases. Information regarding pre-existing medical conditions of the patients from whom the CSF samples were obtained including autoimmune/DM and lymphoma cases.
(TIFF)

S4 Table Disease class, diagnosis, Co-morbidities, age and sex for cases. Information regarding pre-existing medical conditions of the patients from whom the CSF samples were obtained including lymphoma cases.
(TIFF)

S5 Table Key for abbreviations used in heat map and dendrogram. The disease types/classes designated by the abbreviations used in the heat map and dendrogram are provided.
(TIF)

S1 Fig Principal component analysis plot using principal components (PC) 1, 2 and 3. The PCA is based on the following cytokines: EGF, MDC/CCL22, PDGF-AA, Fractalkine/CX3CL1, IFN-γ GRO/CXCL1, IL-15, IL-2, IL-7, IL-8, IL-9, IP-10/CXCL10, TGF-α, IL12-p40, IL12-p70, IL13, IL-1β, and TNF-β.
The key for the cases is shown on the right. Tumor (red), multiple sclerosis (orange), infection (yellow), L = CNS B-cell lymphoma (purple), control (green), autoimmune (blue). This PCA plot demonstrates what analysis of a larger data set might yield in terms of ability of cytokine analysis to separate distinct disease states. In congruence with our initial assessment with the heat map and dendrogram, PCA of our original data set not only demonstrates that cytokine levels are similar among similar diseases, but that certain CNS diseases have relatively predictable cytokine profiles. This is evidenced by the clustering of similar groups when plotted, such as autoimmune disorders, MS, and CNS B-cell lymphomas.
These three disease types form distinct clusters not only as discrete disease groups, but as a class similar to what is seen on the dendrogram (see Fig 1B). The first three components [principal component (PC1, PC2 and PC3)] account for approximately 70% of variation of the data, depicting a fairly comprehensive view of the data. Clustering of the controls (green) is noted, and this cluster lies in the central portion of the graph. A single outlier autoimmune case was a patient with anti-acetylcholine ganglionic neuronal receptor autoimmune encephalopathy. Additionally, there is very close approximation of the three cases of WHO grade III and WHO grade IV gliomas.
Infectious disease represents a very heterogeneous disease group due to the tremendous variety that exists in pathogen classification. The CNS infection cases, therefore, show a generous dispersion favoring the positive aspect of PC1 (horizontal axis) and to the left of PC3 (vertical axis). The few infectious cases in close proximity to the discrete autoimmune, MS, and CNS lymphoma clusters consist of the aforementioned cases of infection in severely immunosuppressed patients.
The extent of clustering identified on this PCA plot supports the potential use of CSF cytokine profiling in distinguishing distinct classes of CNS inflammatory disorders that are frequently difficult to tell apart in the clinical setting.
(TIF)

S1 Data Anonymized data set PONE-D-18-07308R1. Cytokine values (pg/ml) for all clinical cases [C (controls), A (autoimmune disorders), MS (multiple sclerosis cases), LYMPH (lymphomas), GLIOMA (gliomas), and INFECT (infections)] and CSF WBC count (cells/μl), CSF protein (mg/dl) and CSF glucose (mg/dl) data are presented.
(XLSX)
