# Supplementary figures and images for: Potential role of CSF cytokine profiles in discriminating infectious from non-infectious CNS disorders
Source: PLoS One. 2018 Oct 31;13(10):e0205501. doi: 10.1371/journal.pone.0205501 (PMC6209186; doi:10.1371/journal.pone.0205501)

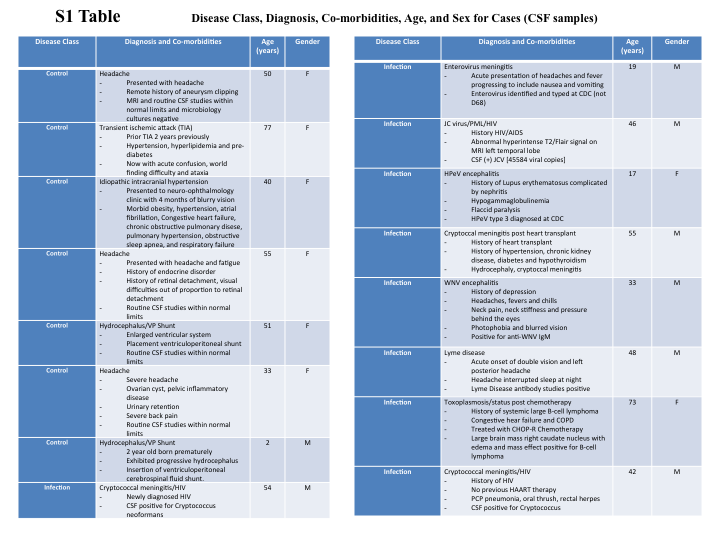

Supplement: S1 Table — Information regarding pre-existing medical conditions of the patients from whom the CSF samples were obtained including control and infection cases. (TIFF) [file pone.0205501.s001.tiff]

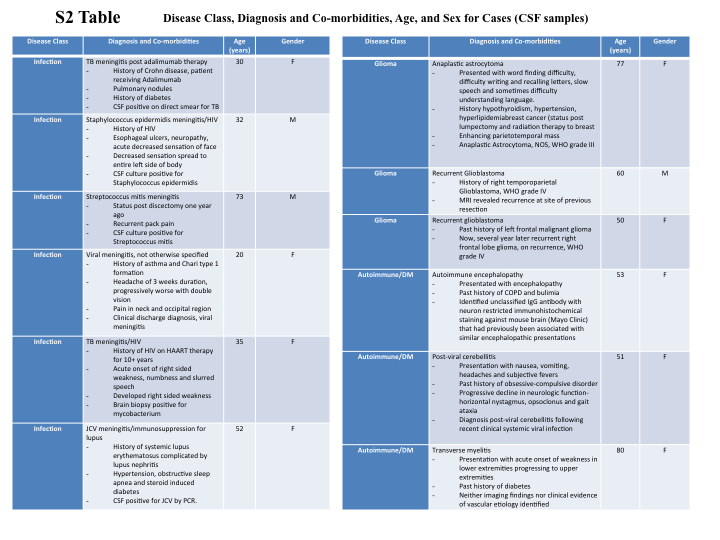

Supplement: S2 Table — Information regarding pre-existing medical conditions of the patients from whom the CSF samples were obtained including infection, glioma and autoimmune/DM cases. (TIFF) [file pone.0205501.s002.tiff]

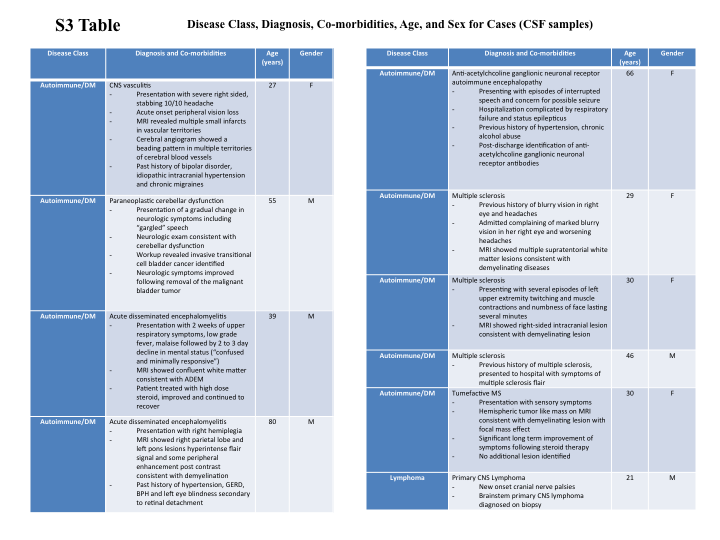

Supplement: S3 Table — Information regarding pre-existing medical conditions of the patients from whom the CSF samples were obtained including autoimmune/DM and lymphoma cases. (TIFF) [file pone.0205501.s003.tiff]

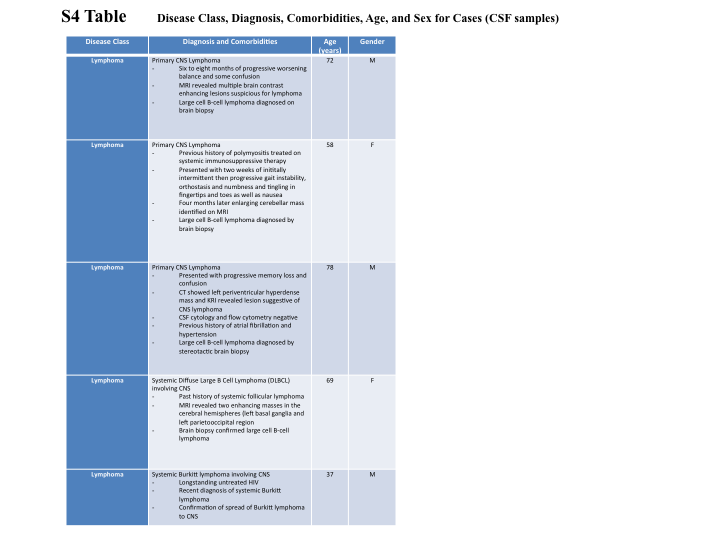

Supplement: S4 Table — Information regarding pre-existing medical conditions of the patients from whom the CSF samples were obtained including lymphoma cases. (TIFF) [file pone.0205501.s004.tiff]

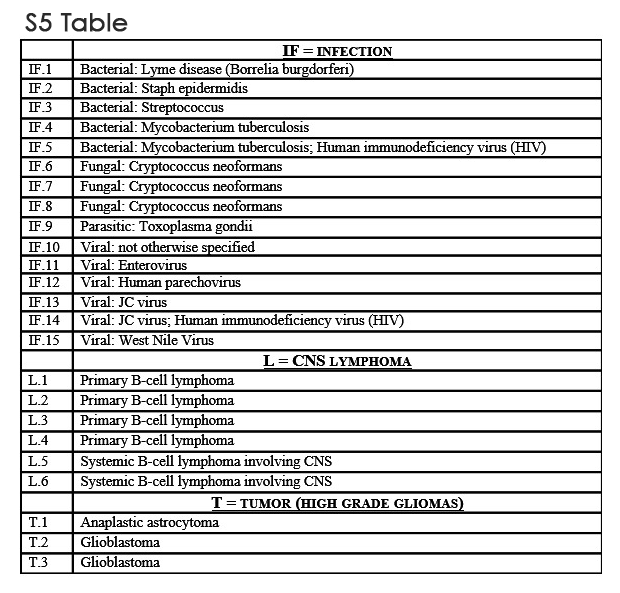

Supplement: S5 Table — The disease types/classes designated by the abbreviations used in the heat map and dendrogram are provided. (TIF) [file pone.0205501.s005.tif]

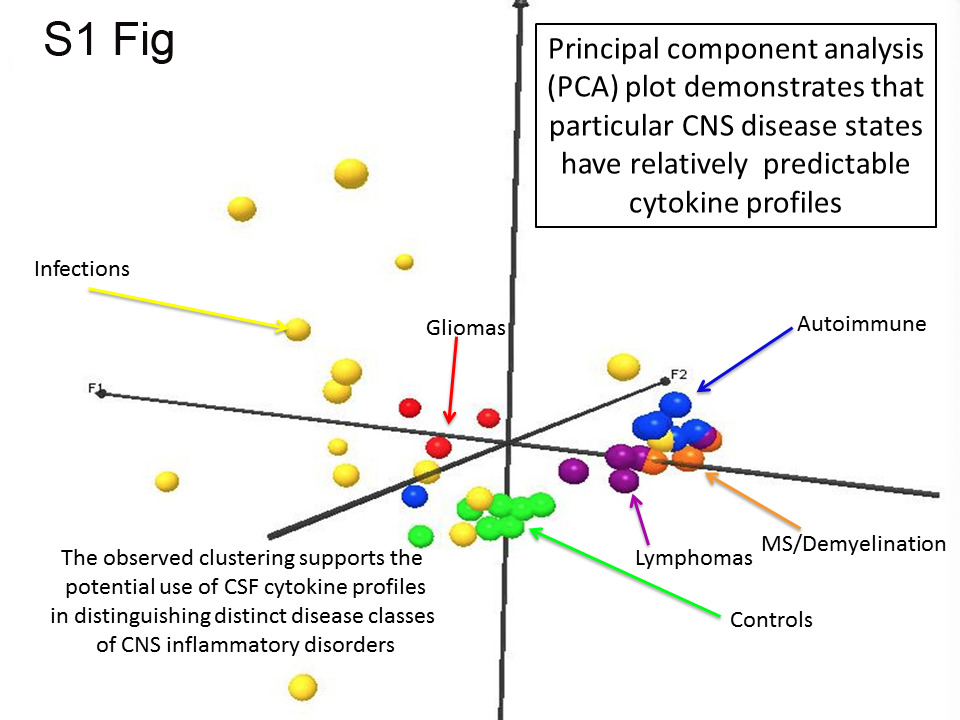

Supplement: S1 Fig — The PCA is based on the following cytokines: EGF, MDC/CCL22, PDGF-AA, Fractalkine/CX3CL1, IFN-γ GRO/CXCL1, IL-15, IL-2, IL-7, IL-8, IL-9, IP-10/CXCL10, TGF-α, IL12-p40, IL12-p70, IL13, IL-1β, and TNF-β. The key for the cases is shown on the right. Tumor (red), multiple sclerosis (orange), infection (yellow), L = CNS B-cell lymphoma (purple), control (green), autoimmune (blue). This PCA plot demonstrates what analysis of a larger data set might yield in terms of ability of cytokine analysis to separate distinct disease states. In congruence with our initial assessment with the heat map and dendrogram, PCA of our original data set not only demonstrates that cytokine levels are similar among similar diseases, but that certain CNS diseases have relatively predictable cytokine profiles. This is evidenced by the clustering of similar groups when plotted, such as autoimmune disorders, MS, and CNS B-cell lymphomas. These three disease types form distinct clusters not only as discrete disease groups, but as a class similar to what is seen on the dendrogram (see Fig 1B). The first three components [principal component (PC1, PC2 and PC3)] account for approximately 70% of variation of the data, depicting a fairly comprehensive view of the data. Clustering of the controls (green) is noted, and this cluster lies in the central portion of the graph. A single outlier autoimmune case was a patient with anti-acetylcholine ganglionic neuronal receptor autoimmune encephalopathy. Additionally, there is very close approximation of the three cases of WHO grade III and WHO grade IV gliomas. Infectious disease represents a very heterogeneous disease group due to the tremendous variety that exists in pathogen classification. The CNS infection cases, therefore, show a generous dispersion favoring the positive aspect of PC1 (horizontal axis) and to the left of PC3 (vertical axis). The few infectious cases in close proximity to the discrete autoimmune, MS, and CNS lymphoma clusters consist of t [file pone.0205501.s006.tif]
